# Effect of Molecular Crowding on DNA Polymerase Reactions along Unnatural DNA Templates

**DOI:** 10.3390/molecules25184120

**Published:** 2020-09-10

**Authors:** Shuntaro Takahashi, Piet Herdwijn, Naoki Sugimoto

**Affiliations:** 1 Frontier Institute for Biomolecular Engineering Research (FIBER), Konan University, 7-1-20 Minatojima-Minamimachi, Chuo-ku, Kobe 650-0047, Japan; shtakaha@konan-u.ac.jp; 2 Medicinal Chemistry, KU Leuven, Rega Institute for Medical Research, Herestraat 49-box 1041, 3000 Leuven, Belgium; piet.herdewijn@kuleuven.be; 3 Graduate School of Frontiers of Innovative Research in Science and Technology (FIRST), Konan University, 7-1-20 Minatojima-Minamimachi, Chuo-ku, Kobe 650-0047, Japan

**Keywords:** molecular crowding, DNA polymerase, unnatural nucleic acids, base pairing, hydrogen bonding, base stacking

## Abstract

Unnatural nucleic acids are promising materials to expand genetic information beyond the natural bases. During replication, substrate nucleotide incorporation should be strictly controlled for optimal base pairing with template strand bases. Base-pairing interactions occur via hydrogen bonding and base stacking, which could be perturbed by the chemical environment. Although unnatural nucleobases and sugar moieties have undergone extensive structural improvement for intended polymerization, the chemical environmental effect on the reaction is less understood. In this study, we investigated how molecular crowding could affect native DNA polymerization along various templates comprising unnatural nucleobases and sugars. Under non-crowding conditions, the preferred incorporation efficiency of pyrimidine deoxynucleotide triphosphates (dNTPs) by the Klenow fragment (KF) was generally high with low fidelity, whereas that of purine dNTPs was the opposite. However, under crowding conditions, the efficiency remained almost unchanged with varying preferences in each case. These results suggest that hydrogen bonding and base-stacking interactions could be perturbed by crowding conditions in the bulk solution and polymerase active center during transient base pairing before polymerization. This study highlights that unintended dNTP incorporation against unnatural nucleosides could be differentiated in cases of intracellular reactions.

## 1. Introduction

DNA and RNA form duplex structures via base pairing. The base-pairing rule in the duplex is based on Watson–Crick (WC) base pairing in living systems. During the transformation of genetic information to the next generation, DNA (or RNA) is replicated by polymerases. Polymerases incorporate the substrate deoxyribonucleotide triphosphate (dNTP) or ribonucleotide triphosphate (rNTP) to replicate DNA and RNA according to the complementarity of the chemical structure of the nucleobase on the template strand. Since polymerases do not look into the chemical structure of the nucleobases of the substrate and template, the matching of substrate and template bases is governed by the thermodynamic stability of the base pairing [1,2]. It has been identified that the energies of hydrogen bonding and base-stacking interactions have a compensatory relationship in the duplex structure [3]. Thus, although the A•T (U) base pair has a smaller hydrogen bond number than G•C, DNA and RNA polymerization could equally occur. The energetic rule for substrate selection also allows non-WC base-pair polymerization, if the energetic gain by hydrogen bonding is enough for efficient and accurate polymerization. These characteristics allow the acceptance of unnatural base pairs in the field of synthetic biology, which will expand the genetic code [4,5,6].

To establish precise polymerization, it is important to examine the possibility of side reactions. Correct base pairing always competes with non-cognate nucleosides during DNA polymerization: A mismatch base pair sometimes stably forms via non-WC hydrogen bonds; however these pairs are generally less stable than WC base pairs [7]. More importantly for biological systems, chemically modified nucleobases can interact with various natural nucleobases via non-WC hydrogen bonding [8]. Furthermore, to apply artificial base pairing in living systems, natural nucleotides can compete with the base pairing of the intended unnatural base pairing [9]. These competitions of natural nucleotides are technically used to manage the risk of engineered bacterial leakage from the laboratory as unnatural genomic base pairs could be replaced with natural DNA after rounds of replication without unnatural base sources [10]. In addition to the modification of bases, the DNA sugar moiety is chemically modified to produce artificial nucleic acids known as XNA, which also show different substrate specificity for natural deoxynucleotide triphosphates (dNTPs) during DNA polymerization [11,12,13]. The understanding of the preference of the substrate against these unnatural (or modified) and natural nucleotides during polymerase reactions is valuable to identify the rule of mutation and redesign the unnatural nucleosides for the efficient incorporation of the intended substrate.

To design novel polymerization base pairs, energy indices about hydrogen bonding and base-stacking interaction have been calculated and used to know how the incorporation of the substrate nucleotides efficiently occurs [2]. However, although substrate nucleotide incorporation is governed by the thermodynamics of base pairing with the template strand, the effect of the surrounding environments on base pairing has not yet been investigated in detail. In living cells, the solution condition is molecularly crowded, which is known as molecular crowding [14]. Under such conditions, the physical properties of the solution are dramatically altered compared with the in vitro experimental condition used commonly. Nucleic acids are among the materials most sensitive to crowding conditions. With the use of crowder molecules such as poly(ethylene glycol)s (PEGs) to mimic intracellular crowding conditions, it has been clarified that DNA duplex thermal stabilities decrease due to a decrease in the water activity [15,16,17]. Molecular crowding also decreases the dielectric constant of the solution, which can increase the ribozyme activities [18,19]. In polymerase reactions, we found that the efficiency and preference of RNA primer extension along the RNA or DNA template by RNA polymerases were drastically affected by molecular crowding [20]: T7 RNA polymerase preferentially incorporated dNTPs over NTPs along the RNA template under specific crowding conditions [20]. Moreover, the fidelity of NTP incorporation was decreased in crowding conditions. These results suggest that the lowering of the dielectric constant promotes both the binding of the substrate to the catalytic center of the enzyme and the formation of base stacking [20]. Based on these findings, it is possible that the primer extension of natural dNTPs against non-cognate unnatural nucleosides by DNA polymerase is largely affected by molecular crowding conditions.

**Figure 1:**
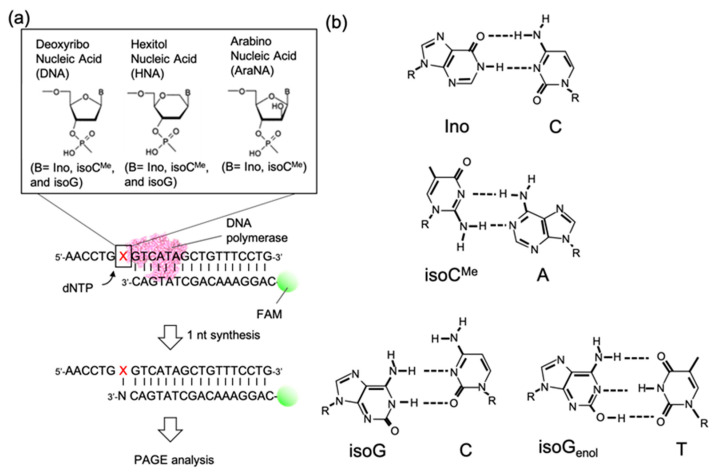
Schematic illustration of this study. (**a**) Setup of the primer extension assay. The “X” in the template DNA indicates the position at which the unnatural nucleoside with various sugar moieties is located. (**b**) Examples of putative base pairs with unnatural nucleobases used in this study and natural nucleobases.

In this study, we investigated the effect of molecular crowding on the efficiency and preference of single primer extension with native dNTPs along a template containing different unnatural bases (inosine: Ino, 5-methyl-isocytosine: isoC^Me^, and isoguanine: isoG) and different sugars (deoxyribonucleic acids: DNA, hexitol nucleic acids: HNA, and arabinose nucleic acids: AraNA) (Figure 1a). Although dNTPs were non-cognate substrate against the unnatural nucleobases on the template, Klenow fragment (KF) DNA polymerase preferred to polymerize a certain dNTP. Interestingly, the trend of polymerization basically indicated the high efficiency of the incorporation of preferred pyrimidine dNTPs with low fidelity but the low efficiency of the incorporation of preferred purine dNTPs with high fidelity. However, in the presence of 20 wt % PEG 200 (average molecular weight 200), the efficiency of the incorporation of preferred pyrimidine dNTPs decreased, whereas that of preferred purine dNTPs increased, resulting in all the efficiencies showing almost similar levels irrespective of the chemical structure of the templates. These findings indicate that preferred pyrimidine dNTPs depend on hydrogen bond formation, which was destabilized by molecular crowding due to a decrease in the water activity. However, the incorporation of preferred purine dNTPs through base-stacking interaction was facilitated by molecular crowding. Our finding suggests that the crowding conditions in a solution and in an enzyme could be key factors determining the efficiency and fidelity of DNA polymerization along unnatural nucleosides.

## 2. Results and Discussion

### 2.1. Design of the Experimental Setup

We performed a primer extension assay to study substrate selection by DNA polymerases under molecular crowding conditions. For the template strand, we designed eight unnatural nucleosides, including three different bases (Ino, isoC^Me^, and isoG) and three different sugar moieties (DNA, HNA, and AraNA) (Figure 1a). Inosine is a natural compound from the deamination of adenine and known to preferentially form a base pair with cytosine (Figure 1a); however, it also possibly forms pairs with other bases via wobble base pairings. IsoC^Me^ and isoG are nucleobases designed to polymerize artificial base pairs using DNA polymerase. However, it is possible to form pairs with natural deoxynucleosides, such as isoC^Me^-A and isoG-C pairs (Figure 1b). The keto-enol tautomerization of isoG also enables stable base pairing with thymine (Figure 1b) [21]. In addition to DNA, we used sugar-modified HNA and AraNA as they are well-known Xeno nucleic acids (XNAs) that could be applied for enzymatic polymerization by DNA polymerase [12,13]. The unnatural nucleoside was placed on the template DNA at the first position to polymerize with dNTPs (Figure 1a). As a primer strand, we used 6-FAM-labeled DNA. As a DNA polymerase, we used the Klenow fragment (KF) derived from *Escherichia coli* DNA polymerase as a standard enzyme. We also examined different DNA polymerases derived from human cells (explained in detail later). For the primer extension assay, preannealed template and primer strands were incubated with a solution containing each DNA polymerase with or without 20 wt % PEG 200 as a crowder molecule and then each dNTP was added to start the single extension of the primer strand. After a 30-min reaction at 37 °C, the product was analyzed via polyacrylamide gel electrophoresis (PAGE).

### 2.2. Primer Extension in the Absence of the Crowder Molecule

**Figure 2:**
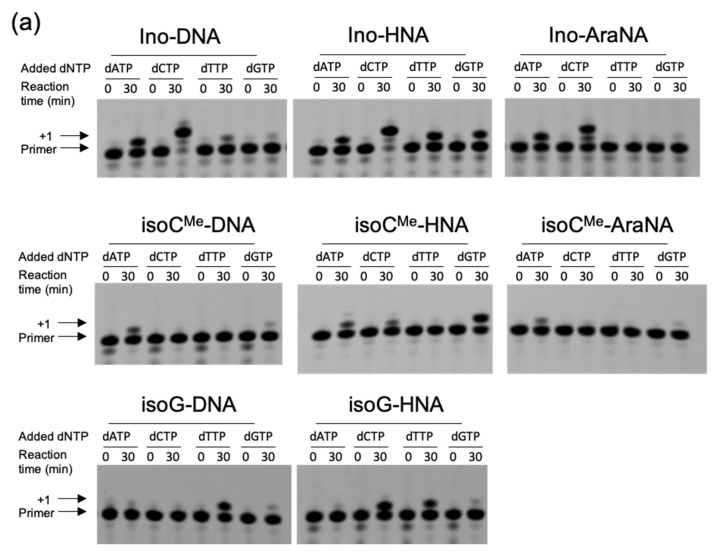

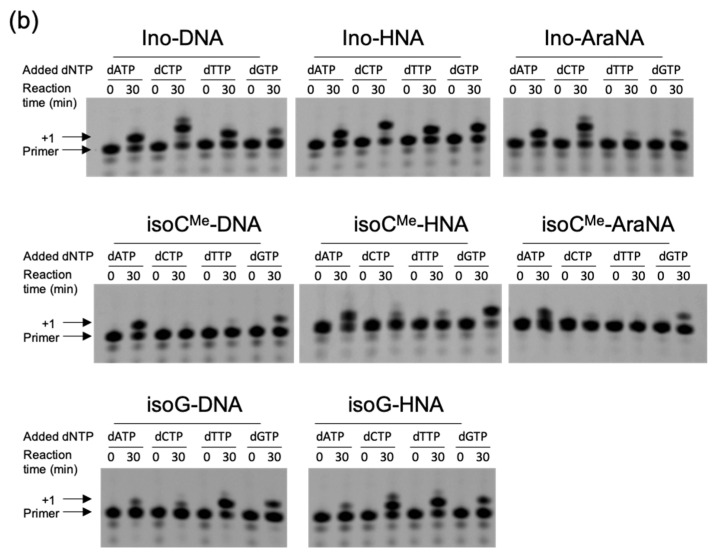
Representative data of polyacrylamide gel electrophoresis (PAGE) analysis of the reactant and products of primer extension by KF in the absence (**a**) or presence (**b**) of PEG200. “+1” indicates the position of the single-base-extended primer. The bands at “+1” and all upper positions were treated as extended products. All the samples were incubated in 10 mM Tris-HCl (pH 7.5), 8 mM MgCl_2_, 100 mM NaCl, and 100 µM of each dNTP with or without 20 wt % PEG 200 at 37 °C for 30 min.

**Figure 3:**
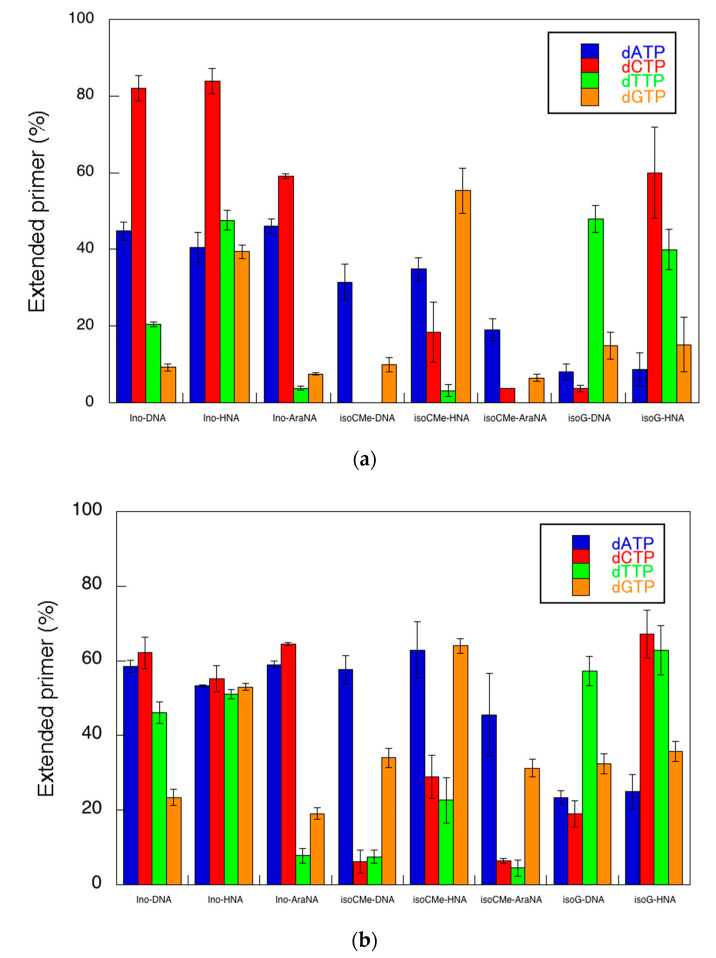
Percentage of extended primers with each dNTP in (**a**) the absence and presence (**b**) of 20 wt % PEG 200.

First, we studied the primer extension by KF in the absence of PEG 200. Figure 2 shows the typical results of the reactions. In the cases of the reactions using the template containing unnatural bases with deoxyribose, KF showed some preferences of substrate selection. To quantitatively analyze the efficiency of the reaction, the percentage of primers extended was calculated as the fluorescence intensity (LAU/mm^2^) of all bands of the extended primer (products showing larger molecular weight than the primer) divided by the summed fluorescence intensities (LAU/mm^2^) of all detectable bands including those of the primer (Table 1). The efficiency of the most incorporated dNTP among the four natural dNTPs was 82.1% with dCTP for the Ino-DNA template, 31.5% with dATP for the isoC^Me^-DNA template and 48.0% with dTTP for the isoG-DNA template, respectively, in the absence of PEG 200. There was a preference for the selection of dNTP in each case, although other dNTPs were also incorporated (Figure 3). For example, although the incorporation of dCTP was preferred for the Ino-DNA template, smaller amounts of other dNTPs were incorporated (dATP: 44.8%, dTTP: 20.4%, and dGTP: 9.3%). Similarly, although dATP was preferred for the isoC^Me^-DNA template, a smaller amount of dGTP (10.0%) was also incorporated. For the isoG-DNA template, dGTP (15.0%), dATP (8.1%), and dCTP (3.7%) were incorporated, although dTTP was preferred (48.0%). These incorporation variety trends were supported by a previous report [12], showing that the deoxy-isoC^Me^ containing duplex stably formed when the complementary base was adenine and guanine, whereas the deoxy-isoG containing duplex stably formed when the complementary base was thymine and guanine.

As shown in Figure 3, the fidelity of the primer extension was also different in each case. In this study, we evaluated the fidelity as the score of preference that indicated how much the most proffered dNTP was incorporated in the reaction. The score of the preference was calculated as the value of the highest efficiency (extended primer (%)) among the substrate dNTPs divided by the summed values of each efficiency (Table 1). According to this calculation, Ino-DNA, isoC^Me^-DNA, and isoG-DNA showed preferences of 52.4%, 75.9%, and 64.5%, respectively. It is important to mention that the efficiency magnitude trend was opposed to the preference, as the order of efficiency is Ino-DNA > isoG-DNA > isoC^Me^-DNA, whereas that of preference is isoC^Me^-DNA > isoG-DNA > Ino-DNA. This relationship might indicate that the efficiency could be dominant by the formation of hydrogen bonding as the fixation of the base orientation by hydrogen bonding should be advantageous for the polymerase reaction in the active site of the polymerase. However, such a hydrogen bonding formation possibility with other dNTPs provides the chance to incorporate with the active site, resulting in low fidelity. However, the fidelity could be dominant by the base-stacking interaction. As the base-stacking interaction shows the complementary relationship with hydrogen bonding [3], the incorporation of bases by stacking dominant manner would decrease the chance of the formation of hydrogen bonding with dNTP, which lowers the efficiency but increases the fidelity.

We also analyzed the polymerization using sugar-modified templates. In the case of Ino-HNA, dCTP was efficiently polymerized as observed in the case of Ino-DNA. However, isoC^Me^-HNA and isoG-HNA preferred dGTP and dCTP, respectively, which were different from those of deoxy types. As shown in Table 1, the efficiencies of polymerization for Ino-HNA, isoC^Me^-HNA, and isoG-HNA were 83.9%, 55.3%, and 60.0%, whereas the fidelities were 40.0%, 49.6%, and 48.7%, respectively. In the case of the AraNA templates, the efficiencies of polymerization for Ino-AraNA and isoC^Me^-AraNA were 59.1% and 19.0%, whereas the fidelities were 50.7% and 68.4% (isoG-AraNA was unable to be used due to the insufficient yield to perform assays). These compensatory relationships were similar to those observed in the case of DNA types. These results suggest that the modified sugars provide a different geometry to the substrate dNTPs that could change the balance of energetic contribution via hydrogen bonding and base-stacking interaction for the polymerization reaction.

**Figure 4:**
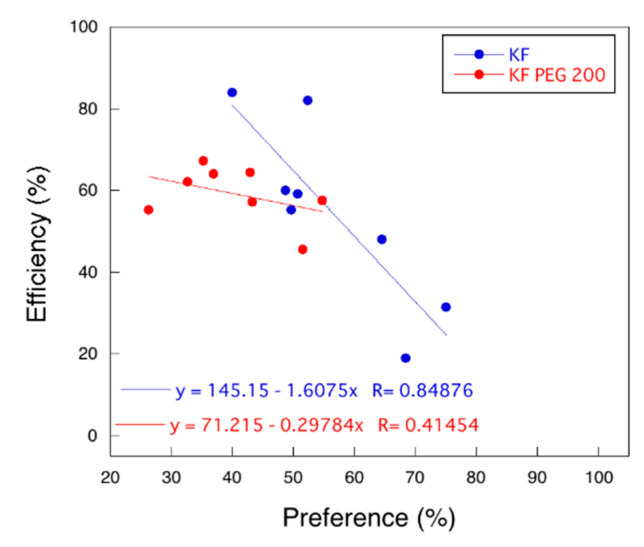
Plots of the efficiency versus preference of the primer extension by KF in the absence (blue plots) and presence of PEG 200 (red plots).

To clearly find the relationship between the efficiency and preference of the primer extension, we plotted the efficiency against the preference of the results above (named the efficiency-preference plot). As shown in Figure 4, the efficiency and the preference are in a negative correlation. This relationship indicates that the correlation could be determined by the energetic balance between hydrogen bonding and base-stacking interaction in the active site of KF, which is irrespective of base and sugar structures.

### 2.3. Primer Extension under Crowding Conditions

Next, we investigated the effect of molecular crowding on the primer extension described above. In this study, PEG 200 was used as a crowder molecule, because PEG 200 is broadly used to study the effect of molecular crowding on not only the stability of DNA duplexes but also polymerase reactions [15,17,20,22]. The decrease in water activity significantly destabilizes the Watson–Crick base pairs [17]. Furthermore, the decrease in dielectric constant results in the enhancement of stacking interactions between the primer and an incorporated nucleotide in the reaction of primer extension using T7 RNA polymerase [23]. PEG 200 decreases these physical factors of the solution more effectively than other crowder molecules such as large MW PEGs. Therefore, we used PEG 200 enabled to perturb hydrogen bonding and base-stacking interactions of the polymerization along the unnatural DNA template. As for the concentration, the effect of crowder molecules on the substrate selection was enhanced when the higher concentration of PEG solution was used [20,23], although the polymerase reactions were inhibited by using PEGs having more than 20 wt % concentrations due to protein denaturation. Thus, we chose 20 wt % as the highest concentration of PEG 200 in the polymerase assays. In the presence of 20 wt % PEG 200, KF also had a polymerase activity (Figure 2b). However, the reactivity was different from that in the absence of PEG 200. Briefly, there are three types of effect of PEG 200 on the primer extension: (Type 1) The efficiencies of templates showing high efficiencies in the absence of PEG 200 decreased. Ino-DNA (82.1–62.1%) and Ino-HNA (83.9–55.2%) were included in this case. (Type 2) The efficiencies of templates showing low efficiencies in the absence of PEG 200 increased. In this case, isoC^Me^-DNA (31.5–57.6%), and isoC^Me^-AraNA (19.0–45.6%) were included. (Type 3) The efficiencies of templates showing middle efficiencies in the absence of PEG 200 showed small change with less than a 10% increment. Ino-AraNA (59.1–64.5%), isoC^Me^-HNA (55.3–64.0%), IsoG-DNA (48.0–57.2%), and isoG-HNA (60.0–67.2%) were included in this case. We have previously reported that the efficiency of the unnatural primer extension using the RNA template by KF was increased in the presence of PEG 200, concluding that the PEG-200-driven dielectric constant decrease facilitated the dNTP binding to the KF active site [20]. The results obtained here were comparable to most cases shown as Type 2 and Type 3 but opposite to Type 1, which indicated that the substrate selection was dependent not only on the decrease of the dielectric constant but also other factors. Interestingly, the favored dNTP in Type 1 was pyrimidine nucleotide (dCTP), while that in Type 2 was purine nucleotide (dATP). These results suggest that the hydrogen bonding which determined the efficiency of incorporation of dNTP was repressed but the base-stacking interaction was facilitated. As a result, the incorporation of purine nucleotide was favored more than that of pyrimidine nucleotide. Although the efficiency was changed in each case using different templates, all the fidelity in the presence of PEG 200 decreased compared with the cases in the absence of PEG 200 (Table 1). Compared with Figure 3a, Figure 3b indicated that the primer extensions with non-preferred dNTPs were increased and the purine nucleotides generally became more preferred than pyrimidine nucleotides. Therefore, in addition to the polymerization enhancement due to the dielectric constant decrease, the driving force of the dNTP incorporation via base-stacking interaction was accelerated more than hydrogen bonding dependent manner.

As shown in Figure 4, the efficiency-preference plot was changed by the crowding condition (red plots in the graph). The trend of the plots was in the negative correlation of the efficiency of the polymerization against the preference. However, the slope of the linear regression of the plots was less than one (0.30) in the presence of 20 wt % PEG 200, whereas it was more than one (1.61) in the absence of PEG 200. The slope means how the reaction occurs efficiently or accurately with various template structure. Thus, the lowering the magnitude of the slope in the presence of PEG 200 quantitatively indicated that the molecular crowding made the polymerase reaction dominant in preference more than efficiency. Although the energies of hydrogen bonding and base-stacking interactions have a compensatory relationship on the duplex structure in the absence of cosolute [3], the results obtained in the presence of PEG 200 imply that the compensatory relationship was changed in the crowding condition. Furthermore, the linear relationships in the presence of PEG 200 suggested that the incorporation of dNTPs was determined by the energetic balance between hydrogen bonding and base-stacking interaction in the active site of KF irrespective of base and sugar structures as observed in the case without PEG 200. Therefore, the drastic change of the efficiency and the preference of the primer extension indicates that molecular crowding could affect the environment of the active site of the polymerase and thus the efficiency and the preference of polymerization were regulated by the environment of the solution.

## 3. Materials and Methods

### 3.1. Materials

dNTPs were purchased from Takara Bio (Japan). PEG 200 and other reagents were purchased from Wako Pure Chemical Industries (Japan) and used without further purification.

### 3.2. Oligonucleotides

All the template DNAs were designed as 5′-AACCTG-X-GTCATAGCTGTTTCCTG-3′. In the position of X, Ino-DNA, Ino-HNA, InoAraNA, isoG-DNA, isoG-HNA, isoGAraNA, isoC^Me^-DNA, or isoC^Me^-HNA were introduced. All the synthesis and purifications were done as previously reported [12]. The HPLC-grade of FAM-labelled DNA primer (5′- CAGGAAACAGCTATGAC-3′) was purchased from Japan Bio Service.

### 3.3. Primer Extension Assay

KF was prepared as an exonuclease deficient type as reported previously [24]. The 50 µM of primer was mixed with an excess of template DNA (100 µM) to completely make the primer strands formed by a duplex. After annealing of the strands, KF (0.1 µM) was incubated with preannealed primer and template strands (5.0 µM), in 10 mM Tris-HCl (pH 7.5) and 8 mM MgCl_2_ 100 mM NaCl with or without 20 wt % PEG 200. After incubation at 37 °C for 1 min, each dNTP (100 µM) was added to start the reaction for 30 min. The reaction was quenched with the addition of 10 mM EDTA and 80% (wt %) formamide. Products were separated by PAGE in a gel containing 20% acrylamide and 8 M urea at 300 V and 70 °C for 2 h in TBE buffer. The gel images were captured using a Fujifilm FLA-5100 fluorescent imager. To quantify the efficiency of the reaction, bands corresponding to extended primers and unreacted primers were quantified by measuring fluorescence intensity as follows: the percentage of primers extended was calculated as the fluorescence intensity (LAU/mm^2^) of the bands of the extended primers divided by the summed fluorescence intensities (LAU/mm^2^) of all detectable bands. For the calculation of the preference, the value of the highest efficiency (extended primer (%)) among substrate dNTPs was divided by the summed values of each efficiency. Data are averages of three samples. Errors are standard deviations.

## 4. Conclusions

In conclusion, molecular crowding could affect the hydrogen bonding and base-stacking interactions in the base pairs of incorporated natural dNTPs and the nucleobase of the unnatural template, which occurs in the active center of reacting DNA polymerases. As a result, the relationship between the efficiency and preference to incorporate dNTPs is altered by the crowding conditions, providing an insight into the incorporation of different dNTPs depending on the solution environment. Furthermore, the environment of the active center is also a factor that affects dNTP incorporation. KF basically incorporates unnatural base pairs dominantly with the ensergetic rules. However, these rules should be different from each polymerase as the enzymes are optimized in the host cell environment. This study could provide potentially useful information about the successful replication of unnatural base pairs under the crowding conditions in certain organelles and the unnatural base pair transformation to the intended natural base pair in the cells.

## Figures and Tables

**Table 1:** Efficiencies and preferences of the primer extension by Klenow fragment (KF) in the absence and presence of PEG200 ^a^.

	Ino-DNA	Ino-HNA	Ino-AraNA	isoC^Me^-DNA	isoC^Me^-HNA	isoC^Me^-AraNA	isoG-DNA	isoG-HNA
In the absence of PEG200								
Efficiency (%)	82.1 ± 3.3	83.9 ± 3.3	59.1 ± 0.6	31.5 ± 4.5	55.3 ± 6.0	19.0 ± 2.8	48.0 ± 3.6	60.0 ± 11.9
(Preferred dNTP)	dCTP	dCTP	dCTP	dATP	dGTP	dATP	dTTP	dCTP
Preference (%)	52.4 ± 0.5	40.0 ± 0.6	50.7 ± 0.9	75.9 ± 2.3	49.6 ± 5.7	68.4 ± 3.5	64.5 ± 4.5	48.7 ± 1.9
In the presence of PEG200								
Efficiency (%)	62.1 ± 4.3	55.2 ± 3.5	64.5 ± 0.4	57.6 ± 3.8	64.0 ± 2.0	45.6 ± 11.1	57.2 ± 3.9	67.2 ± 6.4
(Preferred dNTP)	dCTP	dCTP	dCTP	dATP	dGTP	dATP	dTTP	dCTP
Preference (%)	32.6 ± 0.5	26.3 ± 0.6	42.9 ± 0.7	54.7 ± 2.3	36.9 ± 2.8	51.5 ± 3.6	43.3 ± 2.3	35.2 ± 0.6

^a^ The reaction was performed in 10 mM Tris-HCl (pH 7.5) and 8 mM MgCl_2_ 100 mM NaCl with or without 20 wt % PEG 200 for 30 min.
